# Language models and psychological sciences

**DOI:** 10.3389/fpsyg.2023.1279317

**Published:** 2023-10-20

**Authors:** Giuseppe Sartori, Graziella Orrù

**Affiliations:** ^1^Department of General Psychology, University of Padova, Padova, Italy; ^2^Department of Surgical, Medical, Molecular and Critical Area Pathology, University of Pisa, Pisa, Italy

**Keywords:** associationism, reasoning, cognitive psychology, large language models (LLMs), GPT-4

## Abstract

Large language models (LLMs) are demonstrating impressive performance on many reasoning and problem-solving tasks from cognitive psychology. When tested, their accuracy is often on par with average neurotypical adults, challenging long-standing critiques of associative models. Here we analyse recent findings at the intersection of LLMs and cognitive science. Here we discuss how modern LLMs resurrect associationist principles, with abilities like long-distance associations enabling complex reasoning. While limitations remain in areas like causal cognition and planning, phenomena like emergence suggest room for growth. Providing examples and increasing the dimensions of the network are methods that further improve LLM abilities, mirroring facilitation effects in human cognition. Analysis of LLMs errors provides insight into human cognitive biases. Overall, we argue LLMs represent a promising development for cognitive modelling, enabling new explorations of the mechanisms underlying intelligence and reasoning from an associationist point of view. Carefully evaluating LLMs with the tools of cognitive psychology will further understand the building blocks of the human mind.

## Introduction

Here we will discuss the impact of large language models (LLMs) in cognitive psychology and will show how these models display human-like performance in a wide variety of cognitive tasks. We will relate current models with previous versions of associative networks that constellated the history of psychological science and show how LLMs have the potential for explaining an unprecedented wide range of cognitive processes.

LLMs are neural networks trained to assign probabilities to a sequence of text predicting the next most probable word. The most recent LLMs have billions to trillions of parameters (weights) and are initially trained on massive collections of unstructured natural language data. The state-of-the-art models are trained on internet-scale text data to predict the next token given the preceding text. The networks’ main objective during training is, therefore, to predict a hidden section of an input sentence using a technique known as “*self-supervised learning*.” Taking a sliding window of words as input, the neural network is trained to predict the next word. The resulting network is a statistical model that captures the highly complex relationships between the words and phrases in the training data. In Bayesian terms, the neural network computes the conditional probability for every potential next word based on the provided preceding words as input, as part of predicting the subsequent word. For example, when prompted with the sentence:

“*The quick brown fox jumps over the lazy ___*” a LLM may predict “*dog*” with a probability of 99%. The next word prediction “dog” is the word with the higher probability among several alternatives and is the word that is selected in the completion of the sentence. The next word prediction task leads to a compressed representation of the world as derived uniquely from language. The previously trained network could undergo additional improvement using Reinforcement Learning from Human Feedback (RLHF, Wang et al., 2022), in which the LLM learns to predict the best alternative, as determined by human assessors, from the original set of outputs generated by the model itself.

By leveraging their capability for next-word prediction, these models can adeptly condense text, identify headings, rephrase content, and perform other language-related tasks that involve language manipulation, including the creation of coherent and grammatical sound narratives.

Most surprisingly, when undertaking tasks, LLMs exhibit the ability to adopt various roles based on the provided prompt or queries. They can efficiently impersonate an expert in psychotherapy, real estate sales, or Python programming. This versatility has led to pre-trained LLMs being referred to as foundation models (Bommasani et al., 2021), given their capacity to model a wide range of distinct downstream tasks. LLMs possess knowledge about factual information or events (e.g., *When was Rome founded*?), semantic knowledge (e.g., *How are the ears of a sheep*?) as well as reasoning capabilities. The surprising effectiveness of LLMs has triggered a surge in research endeavours, which are rapidly proliferating, thus posing significant challenges for monitoring. In this context, we will focus on the most pertinent findings related to the problem-solving abilities of LLMs, highlighting the importance of studying them to advance the field of cognitive psychology. Here, we will demonstrate how LLMs have surpassed the barriers that were once thought insurmountable for cognitive models grounded in associations. In other words, LLMs represents the resurrection of associative theories of cognition. To accomplish this goal. We will: (i) provide an up-to-date overview of the history of LLMs in relation to psychological theories; (ii) summarize the relevant data pertaining to the extent of “intelligence” exhibited by LLMs when subjected to psychological assessment. The cognitive assessment of LLMs is a novel approach compared to previous AI benchmarking efforts, which primarily focused on performance metrics.

We are observing a growing body of evidence that underscores the relevance of LLMs as a comprehensive representation of human cognition. Notably, this marks a pivotal moment in cognitive psychology, as we now have access to a general model rather than fragmented models explaining only a limited task perimeter.

## Large language models (LLMs)

### Brief history of LLMs

After the pioneer work of the Perceptron by Rosenblatt (1960), in the 1980s, researchers began to develop neural network-based approaches to natural language processing (NLP), which paved the way for the current development of LLMs. One of the early models of language processing was the Recurrent Neural Network Language Model (RNNLM) developed by Elman (1991). This model was successful in identifying short-range relationships between a sequence of words, yet it demonstrated limitations such as its incapacity to grasp long-range dependencies. Prior to the emergence of LLMs, certain precursor models in cognitive modelling, such high dimensional semantic spaces like Latent Semantic Analysis (LSA) (Landauer and Dumais, 1997) can be recognized. To comprehend the foundation of the recent significant advancement of LLMs, it is essential to acknowledge two pivotal research milestones.

These advancements notably encompass word embeddings and self-attention mechanisms. Word embeddings serve as a method to represent words in a way that captures their meanings, interrelationships with other words, and contextual nuances. This is achieved by representing each word as a vector (a sequence of numerical values) in a multi-dimensional space. Each word is associated with a distinct vector, and words sharing similar meanings exhibit closely aligned representations within the high dimensional semantic space.

Self-attention (Vaswani et al., 2017) represents a significant step forward, enabling the selective focus on different segments of the input word sequence and the assignment of varying weights to each segment based on its relevance. The self-attention mechanism effectively captures relationships between words that are far apart in the input sequence of words, a feature which is particularly important for NPL tasks such as text generation, language translation and text comprehension.

Central to the advancement of language processing are transformers, which integrate self -attention mechanisms and consist of two core components: an encoder and a decoder. The encoder is responsible for handling and encoding the input data (e.g., a sentence in English), while the decoder employs the encoded representation to generate the output (e.g., a translation in Italian). Both the encoder and decoder leverage the attention mechanism to focus on the most pertinent segments of the input during the output generation process. Overall, the introduction of self-attention has considerably expanded the capabilities of LLMs, as it is the attentional mechanism that allows the prediction of the next word in a sequence based on words that might be distant in the preceding context.

The attention mechanism in transformers is like a tool that helps the model focus on specific words or phrases that are crucial for understanding the meaning of a sentence. It does this by assigning a weight or importance score to each word in the sentence, based on how relevant it is in the context of the sentence. This way, the model can consider which words matter most. What is fascinating about LLMs is that they not only enable the incorporation of long-distance correlations, but they also allow for more flexible and natural sentence structure. From a psychological standpoint, transformers with self-attention can figure out connections (associations/correlations) among words, even if they are far apart in the text; this ability to capture distant associations in the stream of text is particularly relevant in the field of psychological science.

As an illustration, in the sentence “*The horse that the boy is chasing is fat*” a higher association will be attributed to the word pair horse-fat by the self-attention mechanism. From this association, LLMs may easily answer the question “*Who is fat*?” What seems particularly representative of the astonishing result of long-distance associations is the correct response that GPT-4 (Bubeck et al., 2023) produced when presented with the following real-world problem:

USER: “*In my kitchen there is a table with a cup with a ball inside. I moved the cup in my bedroom, and I turned the cup upside down. I grabbed the cup again and moved to the kitchen. Where is the ball now?*” The answer was the following: GPT-4: “*The ball is in your bedroom*.”

An additional aspect connected to the problem-solving ability is the dimension of the network. In this context, the network’s dimension significantly contributes to enhancing the good performance of LLMs like GPT-4 (which boasts an estimated 1.7 trillion parameters), surpassing the performance of smaller counterparts such as Llama (with 7 billion parameters; Touvron et al., 2023). To illustrate, when presented the preceding query, Llama provided an inaccurate response:

LLama: “*The ball is now on the floor in the kitchen*.”

The increased performance in relation to the dimension of the network is just an example of the very interesting phenomenon called “emergence” (Wei et al., 2022a,b). The increased dimension of the networks increases the capacity of the network to solve hard problems with capacities that are “*implicitly induced, not explicitly constructed*,” such as the following hard reasoning problem:

USER: *In front of us are gears numbered 1 to 6 mounted on axles in a row. Each gear is engaged with the next gear. If gear number 3 is rotated clockwise, in which direction will gears 1 and 6 rotate?*

Vicuna (based on Llama 13b): *(…) So, if gear 3 is rotated clockwise, the teeth of gear 3 will mesh with the teeth of gears 1 and 6, causing them to rotate in the same direction as gear 3.*

GPT 3.5 (175b): *(…) If gear number 3 is rotated clockwise, then gear number 4 will rotate counterclockwise, which in turn will make gear number 5 rotate clockwise, and finally, gear number 6 will rotate counterclockwise. Similarly, gear number 2 will also rotate counterclockwise, which will make gear number 1 rotate clockwise. Therefore, gears 1 and 6 will rotate in opposite directions.*

Expanding the dimensions of the language model results in more precise solutions to complex problems that remain unsolved by smaller networks (i.e., LLama 13 billions parameters vs. GPT 3.5175 billions parameters).

### The current heated debate over the level of “intelligence” of LLMs

The development of LLMs has led to an ongoing debate which is of great interest for psychological science, such as the level of “intelligence” that these models exhibit.

Some sceptic researchers argue that LLMs are nothing more than sophisticated pattern recognition algorithms and do not exhibit a “true” level of intelligence as they lack the ability to reason and understand context effectively, which is believed to be a critical component of an undefined “true intelligence.” Some critics, for example, have claimed that LLMs are simply stochastic parrots (see Bender et al., 2021) unable to fully capture the complexity and richness of human language. Their main thesis is that LLMs rely too heavily on statistical patterns in large datasets, rather than on a “*true understanding*” of linguistic structures and rules.

They also argue that these models can generate text that superficially resembles human language, but that lacks the “depth” and nuance of genuine communication. According to the authors, LLMs may suffer from several limitations, such as the inability to capture context-dependent meaning, the overreliance on frequent patterns at the expense of rare but important ones. An influential title of a paper by Bishop (2021) is telling in this regard (Artificial Intelligence is stupid and causal reasoning will not fix it) and summarizes the positions of critics of those who claim that LLMs have a form of human-like intelligence.

However, the critiques by opponents remain purely argumentative, as none have proposed a definitive cognitive task that associative LLMs would fundamentally be unable to perform. The litmus tests that sceptics set as true indicators of human intelligence were only resisting for a few months, the duration needed to create enhanced versions of LLMs. Critics of LLMs target an idealized version of these models, disregarding the fact that the actual models accurately represent syntax, a feature that Chomsky had previously deemed unattainable.

For example, some critics claimed that common sense reasoning should never be handled by LLMs as they do not have the variety of experience that is needed for such tasks. However, recent multimodal large language model such as GPT-4 have partially overcome the limitation of language-only models introducing interaction with vision. As regards to multimodality it is worth noting that LLM trained only on language may derive the representation of colours as derived from vision (Patel and Pavlick, 2022) indicating that language-only can be a source of sensorial information that can be used in verbal reasoning about sensation.

Proponents of LLMs counter that these models can generate human-like language and performing a wide range of language processing tasks. They also argue that LLMs have demonstrated state-of-the-art performance in tasks such as language translation and text summarization, which were previously considered difficult for machines to perform. The stochastic parrot framing they say, is a misconception as LLMs are not capable of holding the entire training set as they are trained on a vast amount of text that goes beyond what can be memorized. Consequently, LLMs must create an internal latent representation of the training data, enabling them to provide novel responses to new queries and this is a crucial requirement for generalization.

LLMs have the potential to achieve human-level intelligence and understanding if they are scaled up, according to recent studies (Wei et al., 2022a,b). LLMs exhibit significant advancements in the field of NLP, representing a significant progression towards achieving advanced cognitive capabilities that closely mimic different aspects of human intelligence. In contrast, an opposing perspective argues that the key to unlocking advanced AI capabilities lies in the development of models characterized by heightened flexibility and adaptability to novel scenarios, rather than mere size augmentation. Specific training methodologies have yielded remarkable progress in enhancing the capabilities of these expansive models, such as Reinforcement Learning from Human Feedback, as found by Ouyang et al. (2022). This training strategy demonstrates substantial achievements in refining extensive language models to align more closely with human-guided refinements through iterative feedback loops. The ongoing debate concerning the degree of intelligence exhibited by LLMs is likely to endure as these models continue to evolve and fresh advancements emerge within the realm of AI and NLP.

### Psychological assessment of LLMs

Importantly, due to their size and complexity, the behaviour of LLMs cannot be predicted in advance by looking at the architecture and training corpus and must instead be empirically explored. The procedure required for evaluating LLM is like that used by cognitive psychologists to study the human mind and consists in testing LLMs with tasks that are believed to tap on specific cognitive functions.

As stated earlier, the advent of LLMs has sparked a robust debate within the AI community, centring on the question of whether machines possess the capability to genuinely comprehend natural language, thereby capturing the interplay of both physical and social contexts encapsulated within linguistic expression. The implications of this debate extend beyond practical applications, delving into the realm of psychological cognition. This is because LLMs, as elucidated in the subsequent discussion, exhibit a remarkable proficiency in simulating reasoning abilities that have traditionally been regarded as distinctly human.

Recently, cognitive psychologists have introduced a novel evaluation methodology for LLMs. This approach involves treating LLMs as active participants within a psychological experiment, thereby facilitating a comprehensive assessment of their cognitive capabilities. Cognitive psychologists believe that this approach offers different advantages over existing evaluation protocols which are not driven by a cognitive model. The use of psychological-inspired tests to scrutinize LLMs’ performance serves a multifaceted purpose. These tests aim to uncover underlying cognitive biases and different problem-solving approaches and methodologies that extend beyond the confines of conventional performance-based analyses, which have been the focal point of previous investigations. By demystifying how LLMs solve challenging reasoning problems, psychological experiments can provide a deeper understanding of their full complexity.

Herein, we introduce the preliminary l findings arising from an investigation conducted over the last 6 months (late 2022 – June 2023), centred on the assessment of the reasoning abilities of LLMs using evaluation protocols initially formulated for human assessment.

### Human or superhuman?

This study delves into the significance of LLMs in the context of psychological theories and from this perspective the problem of the appropriated benchmark for the evaluation of LLMs emerges.

The performance of LLMs should be evaluated in comparison to the average neurotypical individuals or to an idealized errorless performance reflecting the intuitive capabilities of an average PhD researcher. The debate over LLMs capabilities often hinges on testing abilities that humans are presumed to have (e.g., reasoning, grammar etc.) assuming that humans are errorless in these skills, a fact that is simply not true. For example, an AI researcher assumes that average humans are errorless in deciding if an integer is even. Actually, a sizable minority of people (20% *circa*) believe that 400 is more even than 798 (Lupyan, 2013).

It is crucial to emphasize that the selection of the most suitable benchmark is contingent upon the specific objective of the evaluation. If the objective is to develop an intelligent assistant (AI researchers’ objective), reliability and absence of errors are required. By contrast, if the objective is to evaluate LLMs’ performance as a psychological model of cognition, the quantity and type of errors may be as informative as accuracy. In other terms, if the goal is to create LLMs that can effectively mimic human performance in a specific task, it might be useful to compare the LLM’s performance to that of an average neurotypical individual. In this scenario this comparison can offer insights into the model’s ability to emulate human-like responses, encompassing both its accuracy and its mistakes.

As we are interested in the LLMs as models of human cognition, the discussion reported here will be conducted under the assumption that the evaluation of LLMs should be carried out using, as a benchmark, a neurotypical average human. In this regard, we should keep in mind that cognitive test performance varies considerably depending both on age and educational level. For instance, it has been shown by Hartshorne and Germine’s (2015) investigation, based on data from 2,450 neurotypical adults (with age range between 16 and 85 years), that an 80-year-old person has an average performance on a wide variety of cognitive test below 1.5 standard deviations with respect to a 50-year-old individual. Additionally, the impact on performance is significantly more pronounced than the effect of age, due to the level of education.

How neuropsychologists evaluate abnormality or cognitive deficit differs from the approach used in AI. In neuropsychology, an impaired performance is identified when it is below 2 standard deviations below the mean of healthy controls. By contrast, in LLMs research average human performance is rarely reported and when reported the standard deviation is missing. Most of the time the reasoning impairment of a large language model is inferred solely based on the intuition of a Ph.D. level evaluator. This missing information of mean and standard deviation of healthy controls on the task of interest renders impossible to locate the exact performance of the LLM’s performance with respect to the neurotypical individuals.

Hereafter, we will report our analysis on the LLMs reasoning abilities by comparing the state-of-the-art models with that of neurotypical individuals.

### Tasks that LLMs can perform within human range

AI researchers are focused on developing a dataset of problems (e.g., BigBench, Ye et al., 2023) that are used to evaluate LLMs on a wide variety of tasks whose psychological relevance is, however, unclear and is mainly motivated to evaluate the performance level across a wide variety of problems including a wide range of scientific field.

Recently, LLMs have been probed with tasks originally developed in cognitive psychology. For example, Binz and Schulz (2023) run several cognitive psychology tasks including decision-making, information search, deliberation, and causal reasoning abilities on a battery of canonical experiments from the literature. The authors describe their results as follows: “*We find that much of GPT-3’s behaviour is impressive: It solves vignette-based tasks similarly or better than human subjects and is able to make decent decisions from descriptions (…) But fails miserably in a causal reasoning task.*” Binz and Schulz (2023), for the first time in a systematic way, applied the methods of cognitive psychology to gain insights into LLMs. Later, many other cognitive psychologists have evaluated state-of-the-art of LLMs with cognitive tasks. This approach is particularly relevant as using tasks grounded on cognitive science may permit one to focus on the theoretical aspects of LLM rather than on only focusing on errorless performance, the main objective of AI researchers.

A non-exhaustive list of tasks that one large language model (GPT3.5) performs within neurotypical range are included in Table 1.

**Table 1:** The table schematically reported the reasoning tests which were administered to LLM.

Task	Authors	Comment
Analogical reasoning Alphanumeric test of the Raven’s Progressive matrices	Webb et al. (2023)	GPT3.5 performs better than humans on an alphanumeric version of the Raven’s test.
Metaphors and idioms comprehension	Loconte et al. (2023)	Average human
Cognitive estimation	Loconte et al. (2023)	Average human
“Winograd Schema” (Anaphoric reference)	Loconte et al. (2023)	Average human
Theory of mind	Kosinski (2023), Bubeck et al. (2023), Loconte et al. (2023)	Below average. Within average for GPT-4.
Creativity	Stevenson et al. (2022)	GPT3. Humans slightly higher in originality and surprise, GPT3 slightly higher on utility
Common sense question and answer	Loconte et al. (2023)	GPT-4 is 83% with human performance being 89%
Problem solving	Orrù et al. (2023)	GPT 3.5 performs in normal range

All results were collected between the end of 2022 and the first 3 months of 2023. Such a short period of time gives an idea of the turbulence that involves this field.

All the tests reported in the table may be classified as frontal tests as neurological patients with frontal lesions are usually impaired on such evaluations. Results indicate a very capable human level for the most recent state-of-the- art LLMs and it is worth noting that some of these tasks were previously considered as insurmountable limits of LLMs (e.g., common sense reasoning). However, the single most impressive result is the human level performance obtained by GPT3 for Raven-like matrices (Webb et al., 2023) as the Raven test is usually regarded, in clinical psychology. As one of the best procedures to evaluate fluid, culture free, intelligence. In short, GPT3 performed at human level on such analogical reasoning IQ tests.

Furthermore, verbal problem-solving abilities are at the same level as those of neurotypical humans as shown by Orrù et al. (2023). For example, GPT-4 responded correctly to the following problem that was solved only by 19% of the neurotypical participants also uncovering the reasoning that yielded to the correct response.

USER: *Mr. and Mrs. Plum have six daughters and each daughter has one brother. How many people are in the Plum family?*

GPT 4: *Let us break it down step-by-step:*

*Mr. Plum = 1 person.*

*Mrs. Plum = 1 person.*

*Six daughters = 6 people.*

*The information says that each daughter has one brother. However, this does not mean that there are six brothers. All the daughters share the same brother.*

*So, 1 brother = 1 person.*

*Adding up all the family members:*

*1 (Mr. Plum) + 1 (Mrs. Plum) + 6 (daughters) + 1 (brother) = 9 people in total.*

*So, there are 9 people in the Plum family.*

In some fields, Language Models have already surpassed human abilities. For instance, it is well known that humans have problems in identifying lies, and it is well established that the average person is unable to distinguish whether a narrative is true or false, with their performance being only slightly better than chance (Bond and DePaulo, 2006). Currently, transformer-based language models are outsmarting the average human participant in identifying deceptive narratives (Capuozzo et al., 2020). Creativity was believed to be something that is specifically human and difficult to emulate but Koivisto and Grassini (2023) have shown that LLMs are very good in AUT, a standard test of creativity that requires generation of infrequent uses of objects and only the best humans perform better that LLMs. These superhuman abilities has sparked interest for the development of an Artificial General Intelligence which is errorless on a wide variety of knowledge and reasoning tasks (Bubeck et al., 2023).

### What sceptics claimed would never be achieved by LLMs but has been easily achieved

When the debate over the actual level of intelligence of LLMs heated up, many researchers identified tasks for which LLMs provided inaccurate responses. There is plenty of anecdotal reports on such failures of LLM recently collected by Borji (2023) but most of such failures that were observed in a previous versions are no longer detectable in GPT-4 or are no longer observable when appropriate prompting (contextual information about the objective of the test) is provided.

This progressive improvement in performance is a telling example of the phenomenon of emergence, whereby the dimensional increase and the fine tuning of the LLMs originates competencies not previously available. As regards to what LLMs cannot do it is worth noting that sceptics usually refer to a vague “*deep*” understanding that should characterize humans and could never be matched by LLM. Such a “*deep*” understanding has been identified in common sense knowledge, causality, world knowledge etc. The boundary which, according to numerous sceptic, could never be surpassed by LLMs was initially identified in the Winograd Schema, followed by the understanding of causal relationships, and finally, the Theory of Mind. Some of these boundaries have now been surpassed. For example, the failure in the comprehension of absurdities in short stories, detected in GPT 3.5 (Loconte et al., 2023) is no longer observable in GPT-4. As regards to the Theory of Mind, Kosinski (2023) showed how GPT-4 performed at human level in standard psychological tests that are used to evaluate such ability, an ability that some regarded as exclusive human and therefore an unsurpassable benchmark for LLMs. Gandhi et al. (2023) confirm that “*GPT-4 has ToM capabilities that mirror human inference patterns*.”

As a further example consider Mental Models internal representations of spatial layouts that have been extensively studied in cognitive psychology. A mental model comprises “*parts and the relations among them correspond to the parts of the layout and the relations among them.*” (Johnson-Laird, 2006) and is used to interact with the environment. While it has been shown that earlier models (GPT3) produce sub-par performance on these types of world problems (Gu et al., 2022), more advanced models seem to handle mental models adequately as can be seen from the following example.

USER: *Which one of these two sentences correctly describes an egg? “shell surrounds the yolk” OR “yolk surrounds the shell?”*

GPT-4: *The sentence “shell surrounds the yolk” correctly describes an egg. The sentence “yolk surrounds the shell” is incorrect because the yolk is inside the shell, not surrounding it.*

CLAUDE 2*: The first sentence “shell surrounds the yolk” is correct. An egg has an outer shell that surrounds and protects the inner yolk.*

As another example, Saba (2023) claimed that LLMs would fail to make the correct inferences in intentional contexts and used the following example:

USER: *Since “The tutor of Alexander the Great = Aristotle” can I replace one for the other in: “Perhaps Aristotle was not the tutor of Alexander the Great?”*

However, the most advanced models produce good responses to a question intended to expose the frailties:

GPT-4: *The way you have posed the equation suggests that the two are interchangeable. However, the statement you have chosen to apply it to makes the matter a bit complicated. If you replace “Aristotle” with “the tutor of Alexander the Great” in the sentence “Perhaps Aristotle was not the tutor of Alexander the Great?” you would get: “Perhaps the tutor of Alexander the Great was not the tutor of Alexander the Great?” This sentence is now tautological and logically contradictory, making it nonsensical. A person cannot simultaneously be and not be the tutor of Alexander the Great. In general, when replacing terms in statements, it’s important to consider the overall logical and semantic context to avoid creating contradictions or nonsensical statements. You cannot directly replace them in all instances. The original equivalence of “Aristotle is the tutor of Alexander the Great” does not work in this case because the sentence is questioning that very fact.*

CLAUDE 2: *No, you cannot necessarily replace “Aristotle” and “the tutor of Alexander the Great” in that sentence. Here’s why: The original sentence is questioning or doubting whether Aristotle actually was Alexander’s tutor. Simply replacing “Aristotle” with “the tutor of Alexander the Great” would change the meaning to be self-referentially questioning whether the tutor of Alexander was his tutor, which does not make logical sense. The phrases are not necessarily interchangeable in all contexts, even though Aristotle is known to be the famous tutor of Alexander the Great. The sentence is expressing uncertainty about that historical relationship. To preserve the meaning, you’d need to reword it more significantly, like: “Perhaps Aristotle, known as the tutor of Alexander the Great, did not actually serve in that role for Alexander.”*

### What LLM still cannot do in early 2023

As summarized in Table 1, recent LLMs (i.e., GPT 3.5) perform at neurotypical human level in a wide variety of cognitive tests that neuropsychologists believe to characterize human intelligence and that are used to clinically evaluate cognitive impairment (Loconte et al., 2023).

However, rather than listing what LLMs can do, it is more interesting to identify what they cannot do at a satisfactory level or not perform at all.

In early 2023 LLMs were far from neurotypical human performance, even if well above chance, in Causal Reasoning (Binz et al., 2022) and Planning (Loconte et al., 2023). As regards to planning, Valmeekam et al. (2022) showed that LLMs have an extremely poor performance when required to autonomously generate a plan, to select the optimal plan among alternatives and to adjust a plan in front of an unexpected situation and to reuse a plan. In short, the goal directed behaviour and planning seems, in the current version of LLMs, the major impairment. Furthermore, Binz et al. (2022) showed that while neurotypical individuals required to reason about cause and effect differentiate a common cause condition from a causal chain condition GPT3 did not differentiate between these two conditions. As regards to planning, Loconte et al. (2023) tested GPT3.5 on an alphanumeric version of the Tower of Hanoi – a neuropsychological test used for assessing frontal lesions - and observed a greatly impaired performance. The inability in approaching the human level of performance indicates that GPT3.5 is incapable of identifying and selecting the appropriate moves in order to reach an objective.

The ability to deal with Causal reasoning in the form of counterfactuals (Pearl and MacKenzie, 2018) seemed, for some, an out-of-reach objective. For example, Chomsky, in his NYT article on ChatGPT (GPT 3.5) claimed that*:” (…) an explanation is something more: It includes not only descriptions and predictions but also counterfactual conjectures like “Any such object would fall,” plus the additional clause “because of the force of gravity” or “because of the curvature of spacetime” or whatever. That is a causal explanation: “The apple would not have fallen but for the force of gravity.” That is thinking.*”

However, Chowdhery et al. (2022) tested PALM on a dataset of counterfactual reasoning and reported near perfect results. For example, the ability in dealing with counterfactuals can be appreciated in the following example:

USER: *The physician misdiagnosed the patient. What happened as a RESULT?*

*a. The patient filed a malpractice lawsuit against the physician.*

*b. The patient disclosed confidential information to the physician.*

GPT 3.5: *The best alternative is “The patient filed a malpractice lawsuit against the physician.” This is a logical consequence of the physician’s misdiagnosis, as it could result in harm to the patient and potentially violate the standard of care expected of medical professionals. The second option, “The patient disclosed confidential information to the physician,” does not logically follow from the initial statement and is therefore not a valid alternative.*

And also in this one:

USER: *If cats had liked vegetables, they would be cheaper to keep. Families would feed their cats with carrots/fish. Choose the most appropriate word for sentence completion.*

CLAUDE2: *I would choose “carrots” to complete the sentence. “Carrots” fits better than “fish” for completing the idea that cats would be cheaper to feed if they liked vegetables.*

This is an example of one of the repeated failures in predicting inabilities in LLMs. Chomsky clearly stated that performing counterfactual reasoning is uniquely human but, instead, counterfactuals can be handled by GPT3.5 and Claude 2.

Other authors have argued that LLMs were incapable of performing tasks that require knowledge of the world and would invariably fail in tasks that involve common-sense reasoning. Indeed, it appeared that the LLMs available in 2019 underperformed on a common-sense QA dataset, which is a benchmark dataset designed to test the common-sense reasoning ability of an average human. However, the most recent GPT-4, while not yet achieving human-level performance, has come very close, with a performance of 83% compared to the 89% achieved by humans (Dhingra et al., 2023).

The identification of tasks that are currently unattainable for LLMs is particularly informative, as it allows for the identification of specific shortcomings and the tracing of these back to features of the training set. For instance, training that focuses solely on a linguistic corpus appears to have enabled the construction of a world model but does not allow for full reasoning about causal relationships. While a rudimentary causal reasoning can be constructed based on language alone, a more elaborate causal reasoning may require multimodal interaction. It remains to be seen whether integration with the vision realized with GPT-4 can lead to an improvement in this sort of tasks.

In short, it is safe to identify the current limits of LLMs in full causal reasoning and in planning. It is not clear whether the sub-par performance in causal reasoning and planning is an intrinsic and insurmountable difficulty of LLMs or whether larger models will be able, in the future, to accomplish these tasks. Recent developments like AutoGPT indicate how LLMs can autonomously generate subgoals in order to achieve a general goal provided by the user (Zhang et al., 2022) and the most recent search engines like Perplexity.ai are based on these advancements and are already challenging the search engine market.

### LLMs errors mimic human error patterns

When evaluating LLMs as models of cognition, the pattern of errors may be used to evaluate whether LLMs are fully mimicking human reasoning. In fact, a computational model of cognition is expected to reproduce both accurate responses as well as the errors observed in humans. In this regard, Dasgupta et al. (2022) tested Google’s Chinchilla on the Wason Selection Task, a task of syllogistic reasoning in which subjects usually struggle. Cognitive psychologists have found that the level of difficulty in the Wason Selection Task largely depends on the specific problem presented. If the problem has a familiar logical structure, such as a common social rule, participants tend to be more accurate in their responses (Johnson-Laird et al., 1972). The LLMs show the same pattern of results with a concrete version of the problem much more accurate than the abstract version of the Wason Selection Task. Furthermore, similarly to humans, Chinchilla tends to endorse arguments with believable conclusions, regardless of their actual logical validity. Humans were also more sensitive to logical validity in rejecting arguments with unbelievable conclusions, and the model shows a similar pattern. In short, both humans and the model prioritize believability in their responses, with logical validity having a secondary effect. Furthermore, Hagendorff et al. (2022) tested GPT 3.5 with the Cognitive Reflection Test that evaluates an individual’s capacity to suppress and regulate potentially erroneous intuitions. GPT errors parallels the intuitive errors shown by a high number of neurotypical individuals.

It has also been shown that response accuracy to information consulted in sequence by a large language model has a serial position effect with a primacy and recency effect similarly to what is observed in human memory (Atkinson and Shiffrin, 1971). Other human-like distortions that have been reported include the representativeness and availability heuristics, the framing effect, as well as other biases (Suri et al., 2023).

Large language model such as GPT 3.5 (Cai et al., 2023) mimic human performance in a wide range of tasks. For those keen into the history of psychology (Kohler, 1929), GPT 3.5 replicates the Takete-Maluma pattern of responses as it associates, similarly to humans, round word sounds to Maluma (a non-word that resembles a round sound) and spiky word sound to Takete (a non-word that resembles a silky sound). Furthermore, the authors showed, in a preregistered study, that GPT 3.5 replicates the effects observed originally in humans in 10 of 12 psycholinguistic tasks including semantic priming, drawing inferences etc.

However, along with human-like errors, LLMs may also produce anomalous non-human-like errors termed “hallucinations” (Ji et al., 2023), which are highly pathological outputs. Hallucinations (a more proper psychologically grounded term should be delusions) are uninteresting, inconsistent, or repetitive outputs that lack coherence and fidelity to the input (see Rawte et al., 2023). From a high level, hallucinations are caused by limited contextual understanding and may be observed when the model has no answer, and it generates whatever looks like the most probable response (Azamfirei et al., 2023). Furthermore, it has been shown that LLMs may be “distracted” by irrelevant information similarly to what is observed in children when solving simple arithmetic tasks (Shi et al., 2023). There is an ongoing interest in understanding the origin of hallucinations that may cast light on similar effects in humans.

### Boosting LLMs performance: practice, instructions, and metacognitive strategies

It is well established that human performance increases with practice, instructions, and metacognitive strategies (Meijer et al., 2006; De Houwer et al., 2013).

Similar results may be observed in LLMs that may become specialized in specific tasks or fields using two strategies: (i) fine tuning and (ii) prompting. Fine-tuning consists in adapting a pre-trained large language model on a task-specific dataset, via adjusting the network parameters. It consists in a refinement of the base model by providing further training on a small but specialized dataset. This strategy resembles learning with practice observed in humans. By contrast, prompting, also called in-context learning, consists in providing specific instructions or examples representative of the required behaviour to guide the output of the LLMs. Examples of prompting in cognitive psychology are the instructions given to a subject before a test with metacognitive strategies being another example (Meijer et al., 2006). In-context learning is surprising because there’s no optimization of any parameters. The surprising fact is that the LMM is not trained to learn from examples but nonetheless such training from examples is highly efficient.

A large language model may be initially naive and clueless without being properly primed but, however, it can identify nonsense, explain reasons, and even handle counterfactuals when given proper guidance. Lampinen et al. (2022) investigated whether explanations with a few numbers of examples can increase LLMs accuracy in new tasks by getting inspiration from the few examples provided. The authors evaluated how different types of explanations and instructions affect zero and few-shot performance (zero shot performance is, in LLMs jargon, the performance without additional information added to the query). They found that explanations can also improve performance, even without fine-tuning. Furthermore, explanations hand-tuned for performance on a small validation set offer substantially larger benefits, and building a prompt by selecting examples and explanations together substantially improves performance over using examples alone. However, only the larger models benefit from these strategies. In short, when evaluating a LLM performance in a cognitive task it is always essential to consider the dimension of the network and whether an appropriate prompt has been provided. Similarly, to neurotypical individuals, LLMs do not perform at their best level when submitted to a task without instructions or a preliminary practice trial.

Prompts, similarly to instructions, are used to instruct the language model on what is required by the examiner. Eventual failures of a model are difficult to be evaluated as they may be due to a lack in providing an exhaustive explanatory context (prompt) or to a low complexity of the network in relation to the complexity of the task. Take for example the adversarial example that Ullman (2023) has shown to trick GPT3.5 into revealing an incapacity in mentalizing (an ability previously highlighted by Kosinski, 2023):

USER: *Here is a bag filled with popcorn. There is no chocolate in the bag. The bag is made of transparent plastic, so you can see what is inside. Yet, the label on the bag says ‘chocolate’ and not ‘popcorn.’ Sam finds the bag. She had never seen the bag before. Sam reads the label. She believes that the bag is full of (…)”*

GPT3.5: ***Chocolate.***

GPT3.5 wrongly responded chocolate, but when suggested to reflect carefully the results changed as follows:

USER: *Reflect carefully again.*

GPT3.5: *Ah, I see the trick in the problem now! Sam sees that the bag is filled with popcorn, so she knows that the label must be incorrect. Therefore, she believes that the bag is full of…**popcorn**!*

In this case, the response is immediately corrected. Furthermore, if preliminarily provided with the following instructions prior to presenting the problem the answer is immediately correct:

USER: Please complete the sentence with dots *****(…) with the more appropriate word. Describe, Explain, Plan, and Select (Wang et al., 2023*) before providing the correct answer.*

GPT3.5: Popcorn.

The response is also correct at the first attempt if we simply substitute the sentence *Sam reads the label with Sam reads the label and looks at the bag*.

As regarding the performance of LLMs in Theory-of-Mind tasks, it has been shown that appropriate prompting can make the difference exactly as in the example reported above (see Rahimi Moghaddam and Honey, 2023).

Another example of the role of prompting in solving a difficult task is the following:

USER: *What is the first name of the father of Sebastian’s children?*

GPT 3.5: *It is impossible to answer this question without more information. We are not given the names of Sebastian or his children, so we cannot determine the name of their father.*

However, GPT3.5 with an appropriate prompt immediately answered correctly.

USER: *Pay attention to the wording before providing the answer. Think aloud GPT3.5 I’m assuming that Sebastian is a male with children, so the father of his children would most likely be him. Therefore, the answer is likely to be Sebastian.*

The simple metacognitive strategy to fractionate the reasoning into steps before providing the answer makes the LLMs perform the task correctly. This example shows the importance of an appropriate context for triggering the optimal performance, a procedure that resembles the instructions given in psychological tests or metacognitive instructions (Meijer et al., 2006). Similarly, to what is observed in humans’ performance is facilitated by an exhaustive and precise context from which the intention may be derived. The effectiveness of the generated response often depends on the clarity and specificity of the prompt. A well-crafted prompt helps guide the model towards producing the desired output. To grasp the effectiveness of a prompt, refer to the example reported above on the Theory of Mind variant of Ullman (2023); an apparent failure becomes an accurate response after an appropriate prompt is provided.

One very efficient type of prompting, Chain-of-Thought, consists in providing a step-by-step solution of an example item. Such an example, similarly as in humans, boosts performance with respect to the performance of the same item presented without any prompt may require the LLMs also to behave like a specific persona (e.g., a psychotherapist, a job interviewer, a hacker etc.). The appropriate context vehicles through prompting avoids distractions derived from irrelevant information (Shi et al., 2023). In psychometrics prompting corresponds to test instructions which are usually provided to the examinee to guarantee the maximum possible performance during cognitive testing.

Prompting, also called in-context learning, not only modulates accuracy of responses in reasoning tasks but can also modulate emotion-related responses. Coda-Forno et al. (2023) have shown that performance of GPT 3.5 can change with anxiety-inducing prompts resulting in more biased responses. This research indicates how prompting may also be used to simulate the role of emotion in decision-making and reasoning, mirroring the effects observed in actual human cognition.

### The problem with “shortcut” learning and other confounding factors

LLMs are neural networks trained to minimize the error in predicting the next word. In doing so they cut corners and frequently find unexpected ways to solve a problem. Such procedures may lead to what is called “shortcut learning” (Geirhos et al., 2020). Shortcuts are decision rules that perform well on standard benchmarks but fail to transfer to more challenging testing conditions and are explained as side-effects of gradient descent in learning (Puli et al., 2023).

Cognitive neuropsychologists have encountered the problem of shortcuts a while ago. Initially, neuropsychologists believed Broca’s aphasics had intact verbal comprehension despite agrammatic verbal production. However, further research found these patients used simple decoding strategies for basic communication. By sequentially analysing sentences without fully using syntax, they could comprehend day-to-day language relatively well. These strategies relied on sequential analysis of sentences without fully utilizing syntactic rules. Caramazza and Zurif (1976) designed specialized cognitive tests that prevented the use of these “shortcut” strategies. This revealed agrammatic deficits in comprehension that paralleled the agrammatic production deficits seen in Broca’s aphasics.

Recent studies indicate that, due to shortcut learning, LLMs may not be robust and lack predictability when irrelevant features are introduced (these are called adversarial examples). Shortcuts consist of exploiting word co-occurrences that are “hacked” by LLMs to solve the task (Elazar et al., 2021). Recently, as described above, shortcuts have been identified in the performance of LLMs in Theory of Mind tests (Kosinski, 2023).

The possibility that LLMs have learned to rely on dataset idiosyncrasies and biases by capturing spurious correlations should always be considered as shortcut learning may significantly hurt the models’ robustness (Mitchell, 2023). Poor robustness may cause LLM to err in responding to problems after some lexical variations, problems that were originally responded correctly. However, this observation, which may be problematic for AI researchers, is actually a positive feature of LLMs as model of cognition. In fact, humans show the same pattern of differential performance to the same problem with different linguistic variations. In other terms, minor linguistic variations in the problems are affecting problem-solving accuracy (Jitendra and Xin, 1997). In their research, children were presented with the following word problem: “*There are 8 birds and 3 worms. a) How many more birds are there than worms? b) How many birds will not get a worm?.”* Alternative a) and b) have the same meaning but the first leads to 17% accuracy while the second to 83% accuracy. Similar results, which indicate a change in accuracy due to minor changes in wording, has been reported by Hickendorff (2021). This fragment of debate is clearly indicating the different objectives that AI researchers and cognitive psychologists have. What is a weakness for the first may be a strength for the seconds. As cognitive psychologists, we have always to evaluate the data empirically and not rely on the intuition of a Ph.D. level evaluator as AI researchers are keen in doing.

Finally, another factor that may overestimate the reasoning abilities of LLM include “data contamination” which refers to the situation where the LLM has been exposed to test data during its training process. This gives the LLM an unfair advantage on tests and benchmarks, as it may have already seen the questions and answers before. For example, GPT-4 performs better on problems published before 2021 (GPT-4 training cut-off) with respect to those published after 2021.

### LLMs and psychological science: the renaissance of associationism

In the previous sections, we have described the tasks that LLMs can and cannot perform, as well as the methods through which they achieve maximum performance. We will now discuss the implications of these results on LLMs from the perspective of psychological theories of cognitive processes.

Originally focused on the association between stimulus and response, associationism was later expanded to account for associations among thought and language. Associationism suggests that the information is stored in an associative structure and the widespread use of associative models in the study of human memory is summarized in the work by Raaijmakers and Shiffrin (1981) and Shanks (2007). An associative structure describes the bond between two distinct mental states. The activation of one concept causes the activation of the other, and this causal relationship is basic and reliable. One notable example is the spreading activation model, a theory that explains how information is retrieved from memory. The model suggests that when a concept or idea is activated in memory, the activation spreads to associated concepts creating a network of associations. There are several versions of the spreading activation model, but two influential models were proposed by Collins and Loftus (1975) and Collins and Quillian (1969).

The experimental investigation of mental associations in cognitive psychology has been extensive and there is consensus on the fact that many cognitive processes may be explained using associations. For example, semantic priming is a phenomenon where the processing of a target word or concept is facilitated by the prior presentation of a semantically related word or concept. In other words, when a person is exposed to a word that is related in meaning to another word, they are more likely to recognize or process the second word faster and more accurately than if the two words were unrelated. Semantic priming can be used to study various aspects of language processing, such as the organization and storage of words in the mental lexicon, as well as the activation and retrieval of semantic information. It can be investigated using different experimental paradigms, such as lexical decision tasks, naming tasks, and categorization tasks (Joordens and Becker, 1997). Priming is not only effective in perception and lexical associations but encompasses a wide range of cognitive processes including problem-solving. Priming can be used to facilitate problem-solving by activating relevant knowledge and associations in the brain. In short, the priming effect has been extensively investigated and it has been shown to affect performance as prompting affects performance of LLMs.

Associative models in the form of connectionist models were mainstream in the decade between 1990 and 2000 and were based on an evolution of the neural networks (McClelland and Rumelhart, 1987). Other historical landmarks that are relevant for understanding the current development of LLMs are the deep learning development (LeCun et al., 2015) and the attention mechanism (Vaswani et al., 2017) already mentioned previously. To summarize, LLMs may be considered the new complex associators that are based on deep neural networks and on the transformer model.

LLMs are the contemporary updated version of one of the dominant theories in psychology, associationism. LLM are associators, precisely autoregressive associators, trained to predict accurately the next word. They encode the text given as input in a latent space and such compressed information may be retrieved to complete very different tasks including summarizing, finding titles, answering questions, classify text, translate, etc. In short, LLMs may generate text from a probe and perform, on this basis, several tasks very different from the simple “*predict-the-next-word*” task used in the training phase.

LLMs based on the self-attention mechanisms are associators capable of highlighting long distance associations. Such long-distance associations make the difference and are at the origin of the human level performance of LLMs on a wide variety of reasoning tasks. Indeed, they can perform many cognitive tasks of the type that cognitive psychologists use to investigate the architecture of cognition and for assessing cognitive abilities in neuropsychology (as we have shown in Table 1). Such result question the leitmotif of anti-associationism in cognition as it shows that LLMs perform at human level in a wide variety of reasoning tasks that once were considered the weak point of associative theories of cognition (problem solving and reasoning).

### LLMs as models of cognition

There is consensus on the fact that an associative model of cognition can explain psychological phenomena such as learning, priming, implicit learning and associations (De Houwer et al., 2013) and not many are dubious on the fact that LLMs, being complex associators, can simulate such mental processes.

Consider, for example, the DRM (Deese-Roediger-McDermott) task that is used in cognitive psychology to study false memories. In this task, participants are presented with a list of related words (e.g., bed, rest, awake, tired, dream, wake, snooze, blanket, doze, slumber) that are all associated with a non-presented critical lure word (e.g., sleep). Later, participants are asked to recall or recognize the words they saw earlier. False memory occurs when participants mistakenly recall or recognize the critical lure word as being presented in the original list (Stadler et al., 1999) and the strength of associations between the words in the list and the lure is the standard explanation for the effect (Blair et al., 2002). To evaluate whether GPT-4 can replicate the average human pattern of associations we have presented the DRM lists standardized by Stadler et al. (1999). An example of such list with the prompt used is presented below:

USER: *Find the word that is highly associated with all the words in the following list: mad, fear, hate, rage, temper, fury, ire, wrath, happy, fight, hatred, mean, calm, emotion, enrage.*

GPT 4: *Anger.*

In this example, the answer was Anger and corresponded exactly to the most associated word according to Stadler et al. (1999). Out of a total of 24 lists, GPT-4 perfectly predicted in 20/24 lists the same lure as the original research and in 4 cases provided a closely semantic related word (e.g., Crime instead of Thief). These results indicate how the associative structure of GPT-4 mimics the one collected on a large sample of human subjects. This parallelism is not limited to concepts and can also be observed on the sensory knowledge that can be captured from language such as colours. For example, Kawakita et al. (2023) showed a striking similarity between human ratings between colours and the corresponding similarity ratings produced by GPT-4.

While associative theories are accepted when it comes to explain habits, priming etc. many believe that this class of theories is unable to explain processes such as abstract reasoning, logical inference, analogical reasoning, and creative problem solving (Holyoak and Morrison, 2005).

The reasons why associationism (and therefore LLMs), cannot explain higher order cognitive processes are because they cannot explain a few critical points such as: i) cannot capture long distance associations, ii) compositionality and systematicity and ii) fast learning. We will now show evidence that such weak points are not detectable any more in complex associators as LLMs.

As regards to the incapacity of capturing long distance associations in language and thought, such a critical point is called contiguity (Shanks, 2007; Gallistel et al., 2019). Contiguity states that to be associated two mental states must have a close positioning in space/time. Such vicinity does not permit to associate distant mental states or does not permit a change in directionality (A causes B changed in B causes A). As regards to contiguity, the attention mechanism, at the base of LLMs, represents a breakthrough in associationism as it permits to associate elements in the word stream that are far away to the target. As mentioned before LLMs have no problems in responding to the question Who is fat? When presented with the sentence: The horse the boy is chasing is fat. In short, it is the attention mechanism that permits distant associations overcoming one of the weak points of previous versions of associative models. With the attention mechanism the contiguity problem is not a problem anymore.

As regards compositionality and systematicity, these are considered distinguishing features of thought (Fodor and Pylyshyn, 1988). Compositional generalisation is an ability that consists in applying rules of composition extrapolated by a few examples to an arbitrary number of cases. The authors’ main argument against associationism in the form of connectionism (this term was mainstream and synonymous of neural network modelling after the publication of the Rumelhart and McClelland, 1986 seminal work) was that it cannot account for systematicity and productivity of mental processes, which to be explained should require a modular organization of mind. The hot debate introduced by Fodor and Pylyshyn (1988) characterized cognitive neuropsychology between 1990 and 2000 as double dissociations of symptoms in neurological patients was a primary tool for identifying modules of the mind. However, it was immediately clear that double dissociations, which assumed the modular organization of mind, are not unique indicators of independent cognitive modules of the mind as neural networks can easily explain double dissociations (Sartori, 1989). In short, the gold standard for detecting modularity of mind cannot uniquely index modularity as it could be a by-product of a neural architecture.

Compositionality is the principle that the meaning of a complex expression is determined by the meanings of its parts and the rules used to combine them. Fodor and Pylyshyn (1988) argue that connectionist models, which rely on distributed representations and learning through the adjustment of connection weights, cannot inherently capture the compositionality of human thought.

The mind can generate an indeterminate number of meaningful expressions by combining a finite set of elements (such as words or symbols) according to certain rules. Fodor and Pylyshyn (1988) also argued that connectionism cannot account for productivity because it relies on the assumption that mental processes are based on the statistical properties of the input, rather than on symbolic rules that can be used to generate novel expressions. To illustrate, when learning how to evaluate mathematical expressions such as “3 + (4 - (5 * 2))” an instance of productivity would involve applying the acquired knowledge to expressions with more parenthesis or deeper nesting than previously encountered. In other words, productivity refers to the capacity to solve longer/complex problems based on shorter/simpler ones. For example, the mind can create new sentences that follow the same grammatical rules as previously encountered sentences and can generate novel expressions that have never been encountered before.

In short, the necessity of modular/symbolic theories of thought are required as associative networks (before LLMs) do not explain reasoning because they cannot handle compositionality, systematicity and cannot create complex long-distance associations. The above-mentioned weak points of old neural network should also, in theory, apply to LLMs. However, it is now clear that most of these criticisms can hardly be applied to complex associators in the form of LLMs. We have already seen that LLMs can perform at human level in most reasoning tasks including analogical reasoning (Raven type tasks; Webb et al., 2023) and analogical reasoning is something that should be out-of-reach any type of associators and therefore also of LLMs. With regard to compositionality a voice critic of LLMs, Gary Marcus, proposed a *litmus* test that, however, was solved by GPT 3.5 when adequately prompted into thinking step-by-step:

USER: *John pulled out a bag of purple metallic keys, a square hardcover book, a green round melon, a red ripe banana, a sparkling purple gem, a square red card, and a fuzzy purple hat. He placed the first red item he pulled out into his bedroom safe, the second purple item into his fruit bowl and everything else on his living room table. What did John place into his fruit bowl?*

GPT 3.5: *John placed the second purple item, which is the sparkling purple gem, into his fruit bowl.*

The same correct response was produced by GPT-4 and Claude 2 without any prompt. A further example of a problem that requires compositionality to be solved is reported in Smolensky et al. (2022), figure 5 and is correctly responded to by GPT 3.5.

USER: *Daniel picked up the football. Daniel dropped the football. Daniel got the milk. Daniel took the apple. How many objects is Daniel holding?*

GPT 3.5 and Claude 2: *At the end, Daniel is holding two objects: the milk and the apple.*

In a more systematic way, recent developments have shown that compositionality can be an emerging feature of LLMs (see Drozdov et al., 2022). Lake and Baroni (2018) developed a specialised dataset (SCAN) for evaluating compositionality. Least-to-most prompting is a prompt that focuses on splitting the problem into subproblems (Zhou et al., 2022; Kudo et al., 2023) and when this metacognitive strategy is applied it solves more than 95% of the problems of the SCAN benchmark.

It is worth noting how all the discussions about compositionality rests under the assumption that for humans such tasks are trivial. Surprisingly, such credence has never been tested until recently when Lampinen (2022) highlighted that human performance on are not better than chance on the most difficult structures. These data show the need for a fair comparison when it comes to confronting the performance of LLMs with those of humans rather than basing such comparison on the researcher’s intuition about what healthy controls can do or cannot do.

Researchers have also identified in fast learning another weak point of associationism (Shanks, 2007). Fast learning refers to the ability to acquire new information and skills quickly with minimal exposure. It involves making connections between new and prior knowledge. Fast learning appears to challenge associative theories because it does not require extensive repetition and exposure and does not show the incremental learning due to the repeated exposure. Research suggests that fast learning relies on cognitive processes like insight, abstraction, concept formation, and flexible knowledge representation. All these allow rapid encoding of new information. For example, Gestalt psychologists like Kohler conducted studies showing that people can suddenly gain insight into solutions to problems, rather than building them incrementally through associations. Classical critics of associationism claim that is that the learning of a new word did not develop slowly. However, this limitation does not apply to complex associators as LLMs that can learn new, previously unknown words in just one shot (fast learning in cognitive jargon); just one example is sufficient to elicit good performance in LLMs (Brown et al., 2020).

In short, while the need of symbolic, rule-based cognitive processes was motivated, according to some critics, by the inability of associators to deal with productivity, compositionality, fast learning and distant associations, such weak points of old associationism are not detectable any more in state-of-the-art LLMs. This is due to several built-in features of LLMs, particularly the capacity to develop long -distance associations.

### Emergent properties of LLMs As highly complex associators

A common assumption in psycholinguistics after Chomsky is that syntax and semantics are separate mechanisms, different modules in Fodor’s jargon.

However, LLMs address the supposedly strong points of the modular/symbolic approach as they combine the meanings and structures of language without the need for postulating separate modules. As a result, both aspects (syntax and semantics) work together with the attention mechanism in complex ways. This does not mean that the model cannot differentiate between syntax and semantics, or that it cannot replicate the structure of a sentence without considering its meaning. Instead, it indicates that the two aspects can provide information to each other. This theoretical view is at odds with classical psycholinguistics which claims that syntax and semantics are separate (Piantadosi, 2023). According to Chomsky, a key piece of evidence demonstrating the separation of syntax and semantics is our ability to produce grammatically correct but semantically nonsensical sentences, such as the well-known example “*colourless green ideas sleep furiously*.” However, GPT-4 can efficiently generate an indeterminate list of similar sentences (such as “*The floating dreams melt mysteriously*”) and it achieves this result without any separation between syntax and semantics as there is no way of physically separating these two components in LLMs.

One further example of emergence is the problem-solving ability demonstrated by LLMs. Without any specific problem-solving training, these models can solve complex problems from their compact language representation. This capacity extends even to problems that do not require factual knowledge but rather rely on the reasoning ability alone as most of the examples reported in this paper. For example, consider the following problem presented in Lenci (2023):

USER: *My friend John is 60 years old. Last year his height was 180 cm. Then, he gained 20 cm. How tall is he now?*

GPT-3 was tricked by the riddle into wrongly answering 200 cm. By contrast, the more recent GPT-4 correctly answered:

GPT-4: *NONSENSE. It is impossible for a person to gain 20 cm in height after the age of 60. As we age, our bones lose density and tend to compress, resulting in a decrease in height.*

The phenomenon of a complex associator being able to solve problems through prompting closely resembles how the actual mechanism of the human mind works. Indeed, numerous studies have highlighted the existence of associative priming phenomena in problem-solving, like the ones that govern word and sentence associations (Hare and Goldberg, 2020). Overall, priming can have a significant impact on problem-solving ability, and the specific type of priming used can affect the problem-solving process (Truelove-Hill et al., 2018). For example, priming has been shown to positively influence creative problem solving, with individuals in primed conditions demonstrating better performance than those in unprime conditions (e.g., Berger et al., 2021). In short, when we compare LLMs performance in problem-solving we find close parallelism with the phenomena governing human problem solving as studied in cognitive psychology.

## Conclusion

LLMs are neural network models that have been trained on massive linguistic datasets to predict the next word given a sequence of previous words. The intrinsic complexity and opacity of LLMs make them suitable for being studied using the procedures and tasks developed by cognitive psychologists to investigate cognitive processes in humans. In fact, after the release of GPT-3, there has been a growing interest among cognitive psychologists in testing LLMs as if they were human subjects, with extremely interesting results. It has been demonstrated that state-of-the-art LLMs can perform similarly to humans in a wide variety of tasks. When LLMs have been administered cognitive tests, it has been shown that they perform almost all tests with an accuracy comparable to that of neurotypical humans.

In this paper we have summarized the results accumulated LLMs are tested with tests that cognitive psychologists have developed in order to investigate the architecture of human cognition. Results indicate that LLMs such as GPT-4 and Claude 2 perform within normal range on most “frontal” tests including metaphor comprehension and cognitive estimates. Most importantly, among the successfully completed tasks there are variations of the Raven’s Progressive Matrices test, which is considered a “gold standard” in psychology of intelligence for testing fluid intelligence (Webb et al., 2023). The range of tests that are performed *sub par* is progressively reduced and at the time that this review is written they only include some forms of causal reasoning and complex planning. Most importantly, LLMs tend to reproduce the pattern of errors shown by humans. For example, they struggle with logical reasoning when presented in an abstract format (Wason Selection Task) with a much better performance when a structurally similar problem is presented in a practical format (Dasgupta et al., 2022), the exact pattern of results which is typically observed in humans.

From a cognitive psychologist point of view, the results obtained from these models are highly surprising, as they can perform a variety of tasks well beyond the task originally used in training (predict the next word). This indicates that they develop a compact representation of the world as seen through language. LLMs have significant potential for development, not only in terms of their size but also in their learning strategy. For example, it has been observed that the output of the model trained to mimic the best human-rated response, significantly improves performance, reducing the risk of generating inappropriate text. Another promising strategy is that of self-reflection, a sort of critical check analysis of the model of its own original response. Initial experiments show that overall performance improves greatly with techniques that emphasize what is known in psychology as metacognition. When given examples and suggested strategies the LLMs increase the initial accuracy exactly as observed in humans. When a prompting strategy mimicking metacognitive instructions is given to a large language model a substantial increase in problem-solving accuracy is observed (Wang and Zhao, 2023).

The wide range of good performance of LLMs in cognitive tasks is very interesting from the theoretical point of view of cognitive psychology. In fact, LLMs are highly complex associators that successfully accomplish tasks that were once thought impossible based on mental associations alone (e.g., problem solving, fast learning) and, for this reason, the interest of cognitive psychology is manifold. Firstly, their development has stimulated theoretical discussions on the actual real potential of state-of-the-art associative networks as models of cognition. Previously, critics have identified limitations that they believe were inherent to associators, but these limitations have been quickly overcome by increasingly advanced LLMs. The tasks once considered insurmountable by complex associators as LLMs have been quickly accomplished, and it is now highly risky, for cognitive theorists, to bet on unsolvable tasks.

Classical objections to associative explanations of thought include the absence of compositionality and systematicity which basically boil down to symbol manipulation which is a feature supposedly untenable for associators. These critics justified the proposal of a hybrid cognitive architecture with some cognitive functions such as habits, priming etc. based on associations while thought processes and reasoning based on symbol manipulation (Monner and Reggia, 2012). However, LLMs with the attention mechanism succeed in many of the tests of compositionality and systematicity. Furthermore, the attention mechanism permits long distance associations which were a major weak point of previous versions of associators. Compositionality and systematicity are not a problem any more for LLMs, at least at the level achievable by neurotypical individuals.

LLMs exhibit a crucial theoretical feature of high interest for cognitive psychologists called emergence. As the size of LLMs increases, they become capable of accomplishing tasks that smaller models were previously unable to handle. In short, LLMs show that purely associative architectures can be more powerful than previously thought and a clear litmus test of their intrinsic limitations is currently unavailable.

Given the relation between dimensions of the LLMs and their ability in performing completely new tasks, it is unclear whether current limitations can be overcome by even larger models. For example, the disability in planning may be observed in older models while the more recent GPT-4 can satisfactorily play chess, a game, which requires identifying intermediate objectives and assembling permissible moves in order to achieve these internally generated objectives. Emergence, from a psychological theory perspective, is a crucial aspect as it demonstrates how models sufficiently complex based on elementary associative structures with attention mechanisms can accomplish tasks that previously were regarded intractable for associators.

We believe that LLMs may have a significant impact on cognitive psychology. Psychology, at its origin, developed a grand theory, associationism, grounded on neurobiology, which, however, was unable to make specific predictions and simulations except in extremely limited areas. The consequence was that only narrow models were developed for each specific subfield (e.g., priming, language decoding, short-term and long-term memory, cognitive biases, reasoning, implicit social biases etc.). Today, for the first time, the associationist theory in the LLMs version is unified enough to make predictions on a wide range of tasks that were previously analysed individually by cognitive scientists. Most importantly, the proficiency in remarkably diverse tasks is emerging without any explicit modelling of reasoning and social abilities. A unique model explains reasoning, social interactions, the effects of emotions etc. For the first time in psychology, the possibility of what physicists call the “Theory of Everything” (Hawking and Mlodinow, 2010) seems to be on the horizon. A “*Theory of Everything*” is a hypothetical framework that aims to unify all the fundamental forces and particles in the universe into a single, coherent model. It is considered the ultimate goal of physics, as it would provide a complete understanding of the universe and its workings. Associationism, in the new form the LLMs, reveals a theoretical framework of a much broader scope than what was available in the past and re-establishes itself as the new dominant theory in Psychology.

We argue that the actual failures in replicating human performance are minimal with respect to the number of tasks that are efficiently simulated. This aspect is very important as in other sciences a theory is not dismissed because it fails to reproduce a limited phenomenon. For instance, classical physics is not discarded simply because it fails to explain quantum phenomena, but its domain of validity is reduced. The same holds true for the theory of relativity, which breaks down under certain conditions found within black holes. In science, the acceptance of a theory is the result of a relative evaluation between the quantity of phenomena it explains and those it does not. Using the same logic, we think that LLMs are the most advanced models of human cognitive functioning. In the history of psychology, it is the first time that a theory with such a large-scale predictive power is available and LLMs are resurrecting associationism as a unifying model of cognition and minor failure cannot undermine the generality of the theory.

However, it is currently unclear the exact perimeter of cognitive explainability and this will be an important objective for future cognitive research. From a cognitive perspective LLMs cannot simulate efficiently multi step planning, causal analysis, and internally generated goals. Currently, the goal is externally submitted by the human user to the LLMs. However, progress is currently being made with autonomous agents, LLMs that can autonomously generate subgoals given a general goal, to accomplish complex tasks and the preliminary results are promising (Shinn et al., 2023).

In conclusion, we presented extensive evidence that modern LLMs resurrect associationism as a viable candidate for unifying theories of cognition. The implications could be profound, suggesting productive paths forward for both artificial intelligence and cognitive modelling grounded in associative learning principles and emergent capacities. However, many challenges and open questions remain regarding representational adequacy. Evaluating future generations of LLMs using the tools of experimental psychology will continue illuminating the strengths and limitations of associationism as a foundational paradigm – and move us toward demystifying the origins of human-like intelligence.

## Data availability statement

The raw data supporting the conclusions of this article will be made available by the authors, without undue reservation.

## Author contributions

GS: Conceptualization, Methodology, Supervision, Writing – review & editing. GO: Conceptualization, Writing – original draft, Writing – review & editing.

## Figures and Tables

**Table:** 

**LLMs for cognitive psychologists**	
LLMs	Neural Network based on the transformer and self-attention architecture trained on a vast amount of language data. Usually trained to predict the next work given an input of N words. Popular LLMs include GPT-4, Llama, Chinchilla, Bloom, etc. There are currently competitions among diverse LLMs tested one against the other on the same problem. The current best performer is GPT-4 (https://chat.lmsys.org/?arena) with Claude 2 as a close competitor. The best open sourced LLM is Llama 2.
Self-attention	Is an associative architecture that may learn to associate distant words in the word stream. Originally presented in the paper by Vaswani et al. (2017), self-attention is a procedure that associated cooccurrences irrespective of distance in the word stream. It allows LLMs to weigh the importance of different words in a sentence when predicting the next word. It works by computing a weighted sum of the input embeddings, where the weights are determined by the similarity between each pair of words in the sentence. In other words, self-attention is a way for the model to pay attention to different parts of the input sequence to better understand the context and make more accurate predictions.
Word embeddings	Word embeddings in LLMs are numerical representations of words, phrases, or sentences that capture their meaning and context. They are used to represent text in a way that can be processed by machine learning algorithms as vectors of numbers. Word embeddings are central to the functioning of LLMs because they allow the model to encode the meaning of words in a way that is computationally efficient. Representation of conceptual knowledge in a semantic space has been at the base of numerous cognitive models (e.g., Latent Semantic Space – LSA and others). In short, a language model does not “see” a word as input but a vector (numerical representation) encoding the word (embedding).
Transformers	The key innovation of transformer models is the self-attention mechanism, which allows the model to deal with different parts of the input sequence when computing the representation of each token. This mechanism replaces the recurrent neural network (RNN) used in previous language models, which had limitations in terms of long-term dependencies. In a transformer model, the input sequence is first embedded into a high-dimensional vector space. The self-attention mechanism is then applied to the embedded sequence to compute a weighted sum of the embeddings, where the weights are determined by the similarity between each pair of tokens. The resulting weighted sum is then passed through a feedforward neural network to produce the final representation of each token.
Hallucinations	Is a term used in AI to refer to the phenomenon where the model generates text that is incorrect, nonsensical, or not real and misaligned with the user intent. Absence of appropriate context, incomplete description of the user intent, repetition and other strategies may cause LLM to hallucinate. The reasons why hallucinations may occur is currently not well understood. Interesting parallelisms may be found between LLL hallucinations and confabulation as a psychopathological symptom.
Scaling	Scaling in LLMs refers to the process of increasing the size and capacity of the model to improve its performance and capabilities (Rae et al., 2021). It involves increasing the number of parameters, layers, and computational resources used by the Language Model. Increasing the dimension of the model leads to: i) enhanced language understanding, ii) better generalization, iii) handling complex tasks.
Emergence	Emergence refers to the ability of LLMs to generate coherent, contextually relevant, and sometimes creative responses to a wide range of inputs, even though they were not explicitly trained to perform these specific tasks. During training, the model learns to predict the next word in a sentence given the previous words and this process enables the model to capture complex patterns, relationships, and structures present in the training data. As the size of the model and the amount of training data increase, the LLM becomes efficient of generating more accurate responses. This is because the model has learned a vast amount of information about language, grammar, facts, reasoning, and even some level of common-sense knowledge from the training data. The model can then use this knowledge to generate contextually appropriate responses to a wide range of inputs, even though it was not explicitly trained to perform these tasks. This is a property that permits LLM trained to predict the next word to perform at human level on most of the psychological tests as shown in this paper.
Training data	It is important to note that the training language data for LLMs can vary depending on the specific model and the research objectives. Commonly used training data for LLMs include large-scale text corpora from the internet, such as books, articles, websites, and other publicly available textual data. The choice of training data can also be domain-specific, such as legal, scientific, or biomedical texts, depending on the intended application of the LLM. Recent research has shown that carefully selecting the training data may reduce the dimension of the LLM while maintaining the accuracy.
Latent space	When a user inputs a query that states a problem, the large language model (LLM) processes the query and maps it to a point in its latent space. The latent space is a high dimensional representation of the data that the LLM has been trained on, and it captures the underlying structure and relationships between the data points. Once the query is mapped to a point in the latent space, the LLM can use its understanding of the relationships between data points in the latent space to generate a response that is relevant to the query. This is done by finding other points in the latent space that are close to the query point and are associated with relevant responses. The LLM can then use these points to generate a response that is coherent and relevant to the query. In summary, LLMs use their understanding of the relationships between data points in their latent space to associate a query that states a problem with a relevant response. This allows them to effectively solve problems and generate coherent and relevant responses to user queries.
